# BC_2_N/graphene heterostructures as anode materials with improved performance for lithium-ion batteries

**DOI:** 10.1039/d5ra07205k

**Published:** 2026-02-05

**Authors:** Jing Zhang, Zhen Yao, Chaoyan Lou, Liming Zhao, Kuixing Ding, Xiongfeng Ma, Wenkai Chen, Pengyue Zhang, Miaogen Chen

**Affiliations:** a College of Science, China Jiliang University Hangzhou 310018 China jingzhang0218@163.com; b Hangzhou Papermate Science &Technology Co., Ltd Hangzhou 310018 China; c Management Science and Engineering, China Jiliang University Hangzhou 310018 China; d College of Materials and Chemistry, China Jiliang University Hangzhou 310018 China; e College of Engineering, Xi'an International University Xi'an 710077 China maxiongfeng1992@163.com; f Department of Chemistry, Fuzhou University Fuzhou Fujian 350116 China wkchen@fzu.edu.cn; g College of Science, Zhejiang University of Science and Technology Hangzhou 310023 China

## Abstract

To meet the increasing demands of the energy storage market, it is imperative to explore and design high-performance anode materials for lithium-ion batteries (LIBs). In this study, we present six types of heterostructures that integrate graphene with BC_2_N-II and BC_2_N-III sheets to explore the electrochemical properties of BC_2_N/graphene systems as potential anode materials for LIBs. Notably, unlike the original BC_2_N-II and BC_2_N-III sheets, which are incapable of adsorbing Li, our findings demonstrate that Li atoms can indeed be effectively adsorbed onto the BC_2_N/graphene heterostructures. Furthermore, the III-HN and III-HH types of heterostructures exhibit significantly enhanced capacity of 414 mAh g^−1^ along with a minimal energy barrier of 0.13 eV. All the evaluated systems exhibit voltages that completely adhere to the current standards for battery anode material applications. This work offers a theoretical framework for designing viable anode materials featuring heterostructures tailored for LIB applications, offering a practical approach to enhance the performance of pristine materials as anodes. This positions BC_2_N-II/graphene and BC_2_N-III/graphene as promising candidates for the future developments of lithium-ion battery technology.

## Abstract 2

To meet the increasing demands of the energy storage market, it is imperative to explore and design high-performance anode materials for lithium-ion batteries (LIBs).

## Graphical abstract

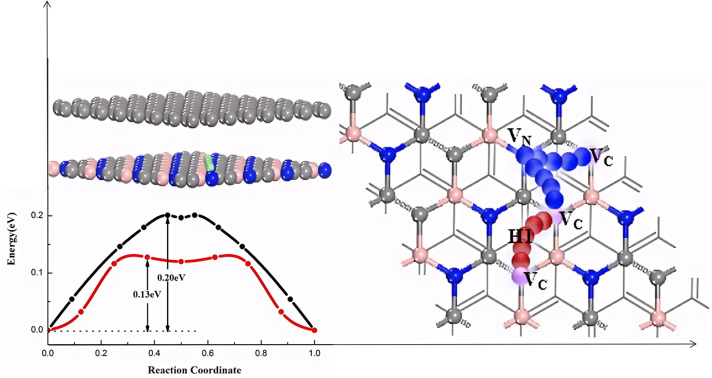

## 1. Introduction

Technologies that use renewable and zero-pollution energy and intermittent electric energy devices have alleviated the pressure brought by the aggravation of environmental pollution and global energy demands.^1–3^ Among these various intermittent energy storage systems, rechargeable lithium-ion batteries (LIBs) are deemed to be promising and attractive due to their high energy density, safety, performance and long cycle life.^4,5^ With the rapid development of commercial portable energy storage devices, the demand for high-performance LIBs has been increasing.^6,7^ As an important component of LIBs, the anode material plays a key role in influencing their performance.^8^ Graphite is widely used as the anode material in conventional LIBs. Currently, the central, critical issue that restricts the further development of LIBs is the low specific capacity and poor charging/discharging rate of the graphitic anode.^9,10^ Thus, to meet the increasing demands of the energy storage market, searching and designing new anode materials for high-performance LIBs is urgent.

One effective way to enhance the performance of LIBs is to explore new materials with large surface-area-to-mass ratios to simultaneously achieve high energy density and ultrahigh charging/discharging rate. Graphene, a two-dimensional (2D) honeycomb carbon monolayer, is considered a potential anode material for LIBs owing to its high surface-area-to-mass ratio (2600 m^2^ g^−1^), superior electronic mobility (10 000 cm^2^ V^−1^ s^−1^) and excellent mechanical strength.^11–14^ Compared with the graphitic anode, graphene monolayer, as an anode, has a stronger ability to absorb Li, affording LIBs with higher capacities. However, one of the major drawbacks of graphene is the weak π–π interaction between its layers,^15,16^ which leads to a decrease in the absorptive capacity of Li, resulting in a loss of capacity in the LIBs. Hence, graphene-like 2D materials, such as hexagonal boron nitride (h-BN), silicene, and C_3_N, have grabbed the attention of the scientific community.^17–21^ Among the family of graphene-like materials, B–C–N materials are heteroatom-substituted carbon systems that are expected to exhibit the hybrid properties of graphene and h-BN monolayers. BC_2_N, as one of the most stable stoichiometric structures of the B–C–N compounds, including BCN, BC_4_N, and BC_6_N, is predicted to have greater potential in exhibiting various physical and chemical properties resulting from their multiple atomic arrangements.^22–27^ Recent studies reveal that the lithium storage capacity of single-layer graphene is diminished compared to that of few-layer graphene. This limitation arises from the enhanced interlayer repulsion forces on both sides of the single sheet, which restrict its Li-ion adsorption capability.^28–30^ Subsequently, few-layer graphene can improve its capacity as an anode material, like the double-layer configuration of graphene (740 mAh g^−1^). However, the experimental capacity of multiple layers has not yet reached the theoretical value of double-layer graphene.^31^ Hence, various heterostructures and bilayer 2D materials have been studied to improve their properties as anode materials for LIBs. Graphene, with its high surface-area-to-mass ratio, is widely used as one substrate of heterostructural frameworks that are combined with other 2D materials to serve as electrode materials. Significantly, it has been shown in extensive theoretical and experimental studies that heterostructure modification is actually an effective way to achieve better performance. Mikhaleva *et al.* found that compared to the VS_2_ monolayer, VS_2_/graphene has higher Li adsorption capacities and can be used as a desirable anode material for LIBs.^32^ This approach has also been applied to MoS_2_/graphene,^33^ MoSe_2_/graphene,^34^ C_3_N/graphene,^35^ WS_2_/graphene,^36^ GeS/graphene^37^ and others.

We selected three possible monolayer geometries of BC_2_N (BC_2_N-I, BC_2_N-II, and BC_2_N-III), which Liu *et al.*^38^ predicted, to investigate the lithium adsorption in three different structures of monolayer BC_2_N in our previous research work.^39^ The theoretical calculations indicate that the adsorption of lithium atoms on the BC_2_N-I monolayer is thermodynamically favorable, whereas the corresponding adsorption processes on the BC_2_N-II and BC_2_N-III monolayers are energetically unfavorable. Furthermore, the BC_2_N-I/G heterostructure system has a larger capacity and a reduced energy barrier compared to BC_2_N-I sheet. Inspired by the remarkable research achievements mentioned above, in this article, we designed six types of heterostructures that integrate graphene with BC_2_N-II and BC_2_N-III to explore the potential of the electrochemical properties of BC_2_N/graphene systems as anode materials for Li-ion batteries with the aim of enhancing the electrochemical properties of monolayer BC_2_N through the construction of heterostructures.

## 2. Computational methods

We performed all density-functional theory calculations using the Vienna *ab initio* Simulation Package (VASP), adopting the projector augmented wave (PAW) approach and the Perdew–Burke–Ernzerhof (PBE) exchange-correlation functional with the generalized gradient approximation (GGA).^40,41^ The semiempirical correction scheme of Grimme (DFT-D2) was adopted to describe the van der Waals energy correction throughout the calculations.^42,43^ For the plane-wave basis cut-off energy, we chose a cut-off energy of 500 eV. The Monkhorst–Pack *k*-point grids of 3 × 3 × 1 and 9 × 9 × 1 were set for the structure relaxation and the electronic structure analysis, respectively. The atomic forces were less than 0.01 eV Å^−1^, and a vacuum space of ∼30 Å was built up to reduce interlayer interactions. In order to calculate the diffusion barrier, we used the nudged elastic band^44^ method. The Bader charge^45^ was chosen to analyze the charge transfer between Li and the sheet.

## 3. Results and discussions

### 3.1. Geometric structures and stability of six possible BC_2_N/graphene heterostructures

**Fig. 1:**
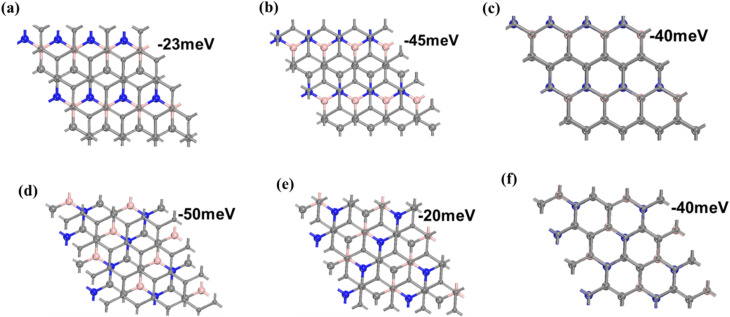
Top views of the (a) II-HN, (b) II-HB, (c) II-HH, (d) III-HB, (e) III-HN, and (f) III-HH heterostructures.

Previous studies^39^ revealed that Li adsorption on the BC_2_N-II and BC_2_N-III sheets is difficult. Thus, we constructed three types of BC_2_N-II/graphene and another three types of BC_2_N-III/graphene heterostructures to investigate the Li adsorption performance. Each type of BC_2_N/graphene heterostructure was constructed using a supercell 2 × 2 of BC_2_N and a 4 × 4 graphene unit cell, along with a negligible lattice mismatch of 0.4%. The heterostructures can be divided into two categories according to the stacking configuration (AA or AB), as presented in Fig. 1. The models defined as II-HN, II-HB, III-HN, and III-HB belong to AB stacking, in which the B or N atoms of the BC_2_N layers and the C atoms of the graphene are right above the center of the graphene and BC_2_N hexagon, respectively. For the models named II-HH and III-HH (AA stacking), the B or N atoms of the BC_2_N sheets are right above the C atoms of the graphene. The stability of the BC_2_N/graphene heterostructures was estimated by calculating the interface formation energy according to the formula:

1 *E*_stack_ = *E*_BC_2_N/G_ − *E*_G_ − *E*_BC_2_N_,

where *E*_BC_2_N/G_, *E*_G_, and *E*_BC_2_N_ denote the total energy of the BC_2_N/graphene heterostructures, graphene, and BC_2_N monolayer, respectively. The stacking energies per carbon atom listed in Table S1 ranged from −20 meV to −50 meV, which is comparable to that of other heterostructures, such as MoS_2_/G^46^ and Blue P/G.^47^ The negative formation energies suggested that the formation of the BC_2_N/graphene heterostructures is an exothermic process, and the heterostructures are stable enough to serve as the anode materials for LIBs.

### 3.2. Li adsorption on the six BC_2_N/graphene heterostructures

**Fig. 2:**
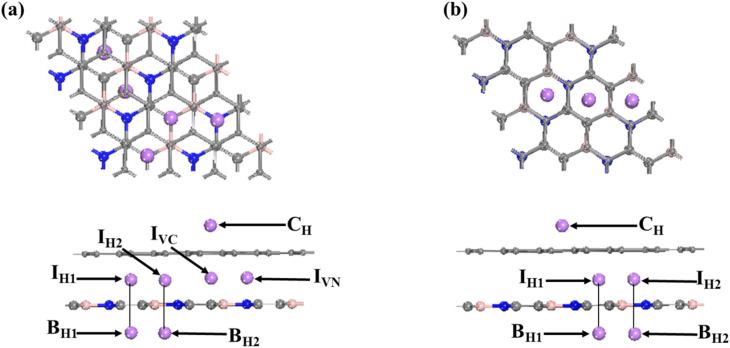
Stable adsorption configurations for Li, including Li/G/BC_2_N (CX), G/Li/BC_2_N (IX), and G/BC_2_N/Li (BX), illustrated in the top and side views of the (a) III-HN and (b) III-HH systems.

Initially, for investigating the Li adsorption behavior on the six possible BC_2_N/graphene systems, we considered three adsorption sites: on top of the BC_2_N surface, in the interlayer of the BC_2_N/graphene, and on top of the graphene. The adsorption energy (*E*_ad_) was calculated as follows:2
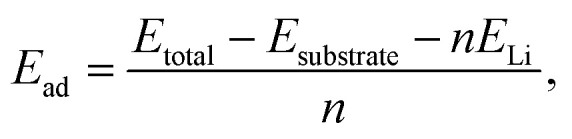
where *E*_total_ and *E*_substrate_ denote the total energies of BC_2_N/graphene with or without the absorbed Li, respectively, and *E*_Li_ represents the energy per atom in metal. Here, *n* corresponds to the number of intercalated Li atoms. According to eqn (2), the adsorption energy was calculated to identify the stable sites of Li adsorption. The preferential adsorption sites and the corresponding adsorption energies for III-HN, III-HH, II-HN, II-HB, II-HH, and III-HB are displayed in Fig. 2, Table 1, Fig. S1, and Table S2. It is exciting to note that in contrast to the original BC_2_N-II and BC_2_N-III sheets, which are unable to adsorb Li, Li atoms can indeed be adsorbed onto the BC_2_N/graphene heterostructures. More specifically, for all six heterostructures, Li embedded in the interlayer of the BC_2_N/graphene (BC_2_N/Li/graphene) shows the largest adsorption energy, followed by Li adsorbed on the top of the BC_2_N surfaces (Li/BC_2_N/graphene) and graphene (BC_2_N/graphene/Li). Hence, Li atoms prefer to be embedded in the interlayers of the systems.

**Table 1:** Calculated adsorption energies (*E*_ad_), Bader charge transfer (*q*), and the distance (height) between the Li atom and monolayer at the stable adsorption sites for Li adsorbed on the III-HN and III-HH heterostructures

System	Li site	*E*_ad_ (eV)	*q* (|*e*|)	Height (Å)
III-HN (BC_2_N/Li/G)	I_H1_	−0.64	0.85	1.57
	I_H2_	−0.51	0.85	1.52
	I_VC_	−0.76	0.84	1.64
	I_VN_	−0.56	0.85	1.65
III-HN (Li/BC_2_N/G)	B_H1_	−0.21	0.88	1.72
	B_H2_	−0.13	0.89	1.72
III-HN (BC_2_N/G/Li)	C_H_	−0.10	0.89	4.91
III-HH (BC_2_N/Li/G)	I_H1_	−1.03	0.85	1.54
	I_H2_	−0.91	0.85	1.55
III-HH (Li/BC_2_N/G)	B_H1_	−0.23	0.88	1.7
	B_H2_	−0.12	0.89	1.74
III-HH (BC_2_N/G/Li)	C_H_	−0.11	0.89	4.82

**Fig. 3:**
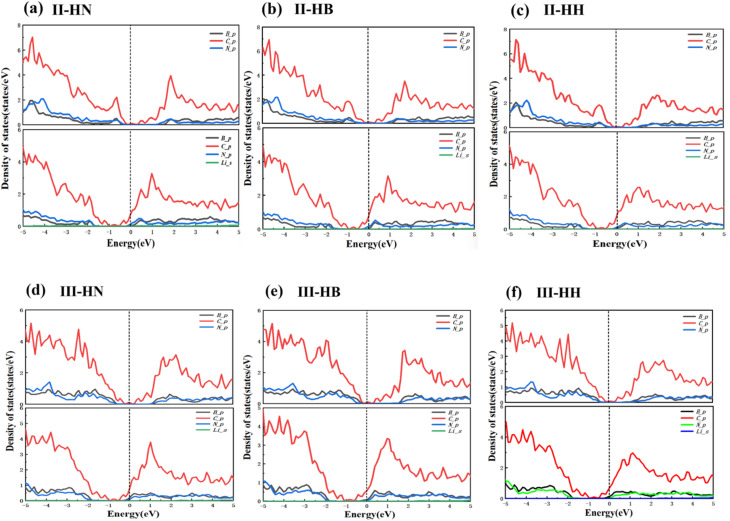
(a)–(f) figures correspond to the projected density of states of the II-HN, II-HB, II-HH, III-HN, III-HB, and III-HH heterfstructures before and after Li adsorption.

To gain further insight into the interactions between Li and BC_2_N/graphene systems, we performed total and projected density of states analyses for lithiated heterostructures, in which the Li atom is embedded in the interlayer with the most stable sites (Fig. 3 and S2). Significant overlaps are observed between the Li 2s/2p orbitals and the 2p orbitals of B, C, and N across the Fermi level, which suggests a strong adsorption relationship between Li and the BC_2_N/graphene system. Meanwhile, the conduction bands shift down, making the systems metallic, which promotes electron transport between Li and substrate systems.

**Fig. 4:**
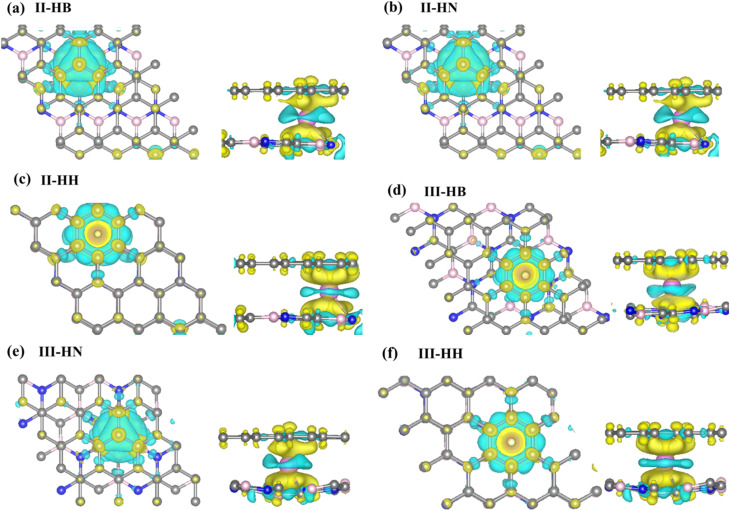
(a)–(f) figures correspond to the charge density difference of the II-HB, II-HN, II-HH, III-HB, III-HN, and III-HH monolayers. Yellow and blue colors indicate charge-accumulated and charge-deficient regions, respectively (the isosurface value is 0.002 e Å^−3^).

Fig. 4 depicts the differential charge density Δ*ρ* of Li located on the most stable sites in the interlayer of the BC_2_N/graphene systems (BC_2_N/Li/graphene). As shown in Fig. 4, a net loss of electronic charge is found around the Li atoms, and an accumulation of electronic charge is found around the interlayer of the systems, which indicates a certain amount of electron transfer from the Li atom to both the BC_2_N and graphene layers. Large-scale electronic transfer leads to strong ionic bonding between the embedded Li and the BC_2_N/graphene layers. Bader charge analysis reveals a significant electron transfer of approximately 0.84–0.90 *e* from Li to the BC_2_N/graphene (Tables 1 and S2), corresponding to the formation of positively ionized Li species. This charge-transfer-induced compensation is a key factor stabilizing the adsorption. Both this quantification and the differential charge density (Δ*ρ*) results collectively confirm the strong ionic character of the interaction between Li and the substrate.

### 3.3. Li diffusion on the six possible BC_2_N/graphene heterostructures

**Fig. 5:**
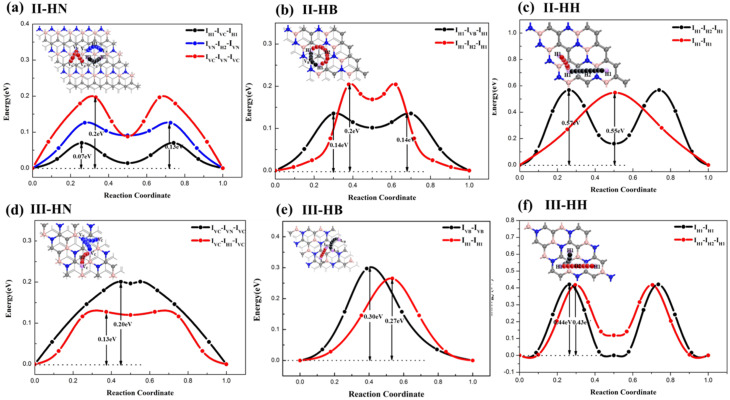
(a)–(f) figures correspond to the considered diffusion pathways and the corresponding energy barriers for six possible BC_2_N/graphene heterostructures.

The diffusion behavior of Li on the six possible BC_2_N/graphene heterostructures was also investigated. Since the most favorable sites of Li insertion in the six systems are all in the middle layer, we only present the diffusion pathways of lithium atoms embedded in the heterostructures. The mobility (diffusion pathway) of Li from one stable site to the adjacent next equivalent stable site is depicted in Fig. 5. The lowest diffusion barrier is 0.07 eV, which is higher than that at the interface of graphene and blueP in the graphene/blueP/MoS_2_ system (0.066 eV),^48^ but lower than those of BC_2_N-I sheet (0.24–0.68 eV), I-BN (0.073–0.435 eV), and I-HH (0.470.73 eV) and the defective BC_2_N systems (0.252.0 eV) presented in our previous research work.^39^ Among the heterostructures, II-HN, which has three diffusion pathways, possesses the most preferable diffusion pathway (I_H1_–I_VC_–I_H1_) with the lowest barrier of 0.07 eV in all of the systems. This diffusion barrier of Li in the interlayer is lower than that of most reported heterostructure materials, such as C_3_N/graphene (0.28 eV)^35^ and blueP/graphene (0.15 eV).^47^ Similarly, II-HN also has two other diffusion paths with ultra-low energy barriers (no more than 0.20 eV). For III-HB, the diffusion barriers are 0.27 and 0.30 eV, which are lower than those on SiC/graphene (0.63 eV),^49^ MoB_4_ (0.54 eV),^50^ or CrB_4_ (0.52 eV),^50^ or the pristine graphene monolayer (0.32 eV),^51^ MoS_2_ (0.22 eV),^52^ or SnC(0.33 eV).^20^ II-HH and III-HH have two diffusion pathways with higher barriers of 0.55–0.57 eV and 0.43–0.44 eV, respectively.

### 3.4. Theoretical storage capacity and average voltage

Beyond electronic structures and ionic diffusivity, the storage capacity and open-circuit voltage constitute two further critical evaluation parameters for LIB anode materials. To this end, the maximum lithium storage capacity (*C*) is estimated as follows:3
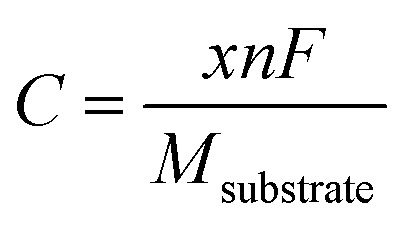
Here, *x* represents the concentration of Li atoms within the substrate, and *n* is the associated electronic charge number. The Faraday constant *F* is taken as 26 800 mAh mol^−1^, and *M*_substrate_ denotes the molecular molar mass of the substrate per formula unit. In addition, the open-circuit voltage (OCV) is estimated by using the following equation:4
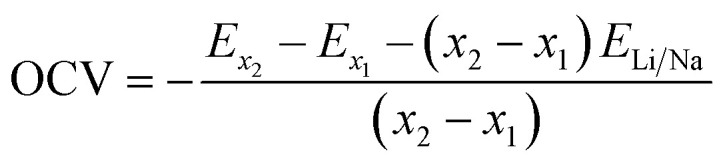


**Fig. 6:**
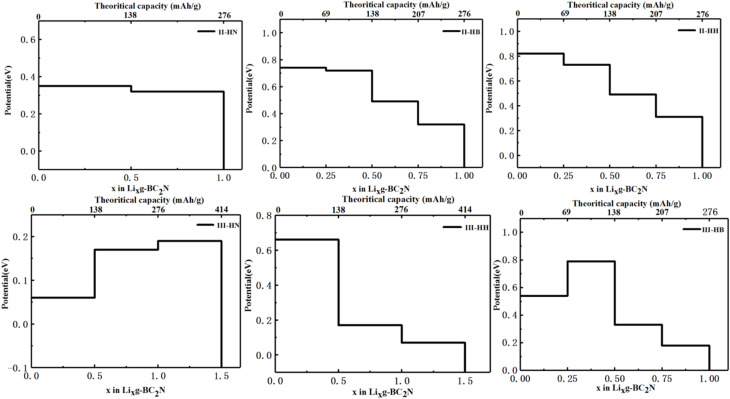
Open-circuit voltages and the calculated storage capacities of six types of BC_2_N/graphene heterostructures: II-HN, II-HB, II-HH, III-HB, III-HN and III-HH.

In this expression, *E*_*x*_2__ and *E*_*x*_1__ refer to the total energies at adjacent Li concentrations (*x*_2_ and *x*_1_), respectively, and *E*_Li/Na_ is the energy per atom in the corresponding bcc lattice. To determine the maximum Li adsorption capacity, Li atoms were iteratively added to the system's most stable site. The loading process was continued until the system's Gibbs free energy turned positive or a significant structural distortion indicated instability, marking the saturation point. The corresponding open-circuit voltages and storage capacities are presented in Fig. 6. The average voltage is determined by the overall change in Gibbs free energy (Δ*G*_f_) for the adsorption process; it is calculated according to the relation *V*_avg_ = −Δ*G*_f_/Δ*xe*, where Δ*x* is the change in Li content and *e* is the elementary charge, and Δ*G*_f_ = Δ*E*_f_ + PΔ*V* − *T*Δ*S*. With volume and entropy effects both neglected,^53^*V*_avg_ = −Δ*E*_f_/Δ*xe*.^54^ When the systems reach the maximum adsorption capacity, we can find the maximum lithium intercalation concentration and the Δ*E*_f_ value before the substrates adsorb Li and after the substrates reach the maximum lithium intercalation concentration. Therefore, the average voltages of six BC_2_N/graphene heterostructures were calculated. Remarkably, the BC_2_N/graphene heterostructures exhibit significantly enhanced Li storage capacities. This stands in marked contrast to the pristine BC_2_N-II and BC_2_N-III monolayers, which themselves demonstrate negligible Li adsorption capability. Especially, in the BC_2_N/graphene heterostructures, III-HN and III-HH have higher storage capacity (414 mAh g^−1^), with average voltages of 0.32 and 0.36 eV, respectively. The average voltages of II-HN, II-HB, II-HH, and III-HB were calculated to be 0.53, 0.57, 0.59, and 0.46 eV, respectively, with the same theoretical capacities of 276 mAh g^−1^. The calculated voltages for all systems fall within the target ranges of 0.1–0.0 eV for high-performance LIB anodes. It should be noted that the theoretical capacities of the BC_2_N-II/graphene and BC_2_N-I/graphene heterostructures are greatly improved compared to those of pristine BC_2_N-II and BC_2_N-III monolayers, lower than those of BC_2_N-I (547 mAh g^−1^)^39^ and I-BN (690 mAh g^−1^),^39^ the same as that of I-HH (414 mAh g^−1^),^39^ but higher than those of stanene (226 mAh g^−1^),^55^ Mo_2_C (146 mAh g^−1^).^56^

## 4. Conclusion

In this study, we have performed a comprehensive theoretical investigation into the performance of six types of heterostructures that combine graphene with BC_2_N-II and BC_2_N-III sheets as anode materials for lithium-ion batteries, utilizing first-principles calculations. The adsorption and diffusion behavior of lithium atoms on BC_2_N/graphene heterostructures has been thoroughly investigated. For the BC_2_N monolayer, which is unable to adsorb Li atoms, the BC_2_N/graphene heterostructures demonstrate a stable capacity for Li atom adsorption. Moreover, we found that the III-HN and III-HH heterostructures have greatly improved capacities of 414 mAh g^−1^, with the lowest energy barrier of 0.13 eV. The calculated voltages for all the systems satisfy the key performance criteria for LIB anodes, falling within reported desirable ranges. This is enabled by the heterostructure design, which, as electronic structure analysis reveals, provides an effective mechanism to tailor the electronic properties and enhance the performance of pristine BC_2_N-II and BC_2_N-III monolayers. Our findings present a practical route to overcome a key limitation of pristine materials, advancing their development as high-performance anodes for LIB applications.

## Author contributions

Jing Zhang: methodology, software, writing – original draft. Wenkai Chen: supervision, funding acquisition, conceptualization. Xiongfeng Ma: writing – review & editing. Zhen Yao: data curation. Chaoyan Lou: formal analysis. Liming Zhao: software. Kuixing Ding: formal analysis. Pengyue Zhang: funding acquisition, conceptualization. Miaogen Chen: review, editing, conceptualization.

## Conflicts of interest

The authors declare no competing financial interest.

## Supplementary Material

RA-016-D5RA07205K-s001

## Data Availability

Data will be made available on request. Supplementary information: detailed results providing additional information for Fig. S1, top and side views of stable adsorption sites for Li-ion adsorption in Li/G/BC_2_N, G/Li/BC_2_N, and G/BC_2_N/Li of II-HN, II-HB, II-HH, and III-HB. Fig. S2, total densities of states of II-HN, II-HB, II-HH, III-HN, III-HB, and III-HH heterostructures before and after Li adsorption. Table S1, calculated the formation energies (*E*_stack_), as well as the total energy of the BC_2_N/graphene heterostructures (*E*_BC_2_N/G_), graphene (*E*_G_), and BC_2_N monolayer(*E*_BC_2_N_). Table S2, calculated adsorption energies (*E*_ad_), Bader charge transfer (*q*), and the height between Li atom and monolayer at the more stable adsorption sites, for Li adsorbed on II-HN, II-HB, II-HH, and III-HB heterostructures. See DOI: https://doi.org/10.1039/d5ra07205k.

## References

[cit1] Armand M., Tarascon J. M. (2008). Nature.

[cit2] Suh S. (2006). Environ. Sci. Technol..

[cit3] Van Noorden R. (2014). Nature.

[cit4] Etacheri V., Marom R., Elazari R., Salitra G., Aurbach D. (2011). Energy Environ. Sci..

[cit5] Goodenough J. B., Park K. S. (2013). J. Am. Chem. Soc..

[cit6] Dunn B., Kamath H., Tarascon J. M. (2011). Science.

[cit7] Chen H., Cong T. N., Yang W., Tan C., Li Y., Ding Y. (2009). Prog. Nat. Sci-Mater..

[cit8] Guo G. C., Wang D., Wei X. L., Zhang Q., Liu H., Lau W. M., Liu L. M. (2015). J. Phys. Chem. Lett..

[cit9] Dahn J. R., Zheng T., Liu Y. H., Xue J. S. (1995). Science.

[cit10] Yang S., Feng X., Ivanovici S., Muellen K. (2010). Angew. Chem., Int. Ed..

[cit11] Novoselov K. S., Geim A. K., Morozov S. V., Jiang D., Zhang Y., Dubonos S. V., Grigorieva I. V., Firsov A. A. (2004). Science.

[cit12] Xu C., Xu B., Gu Y., Xiong Z., Sun J., Zhao X. S. (2013). Energy Environ. Sci..

[cit13] Stoller M. D., Park S., Zhu Y., An J., Ruoff R. S. (2008). Nano Lett..

[cit14] Geim A. K., Novoselov K. S. (2007). Nat. Mater..

[cit15] Ling C., Mizuno F. (2014). Phys. Chem. Chem. Phys..

[cit16] Li X., Hu Y., Liu J., Lushington A., Li R., Sun X. (2013). Nanoscale.

[cit17] Shukla V., Araujo R. B., Jena N. K., Ahuja R. (2017). Nano Energy.

[cit18] Yu X., Chen X., Wang X., Yuan Z., Feng J., Rong J. (2021). Chem. Eng. J..

[cit19] Bhauriyal P., Mahata A., Pathak B. (2018). J. Phys. Chem. C.

[cit20] Rehman J., Fan X., Zheng W. (2021). Mater. Today Commun..

[cit21] Kim K., Choi J. Y., Kim T., Cho S. H., Chung H. J. (2011). Nature.

[cit22] Zhu Y. W., Murali S., Stoller M. D., Ganesh K. J., Cai W. W., Ferreira P. J., Pirkle A., Wallace R. M., Cychosz K. A., Thommes M., Su D., Stach E. A., Ruoff R. S. (2011). Science.

[cit23] Kaner R. B., Kouvetakis J., Warble C. E., Sattler M. L., Bartlett N. (1987). Mater. Res. Bull..

[cit24] Kouvetakis J., Sasaki T., Shen C., Hagiwara R., Lerner M., Krishnan K. M., Bartlett N. (1989). Synth. Met..

[cit25] Belasfar K. (2020). J. Phys. Chem. Solid.

[cit26] Besmann T. M. (1990). J. Am. Ceram. Soc..

[cit27] Ottaviani B., Derre A., Grivei E., Mahmoud O. A. M., Guimon M. F., Flandrois S., Delhaes P. (1998). J. Mater. Chem..

[cit28] Pollak E., Geng B., Jeon K. J., Lucas I. T., Richardson T. J., Wang F., Kostecki R. (2010). Nano Lett..

[cit29] Fan X., Zheng W., Kuo J. L. (2012). ACS Appl. Mater. Interfaces.

[cit30] Fan X., Zheng W. T., L. J., Singh D. J. (2013). ACS Appl. Mater. Interfaces.

[cit31] Hu W., Wang T., Zhang R., Yang J. (2016). J. Mater. Chem. C.

[cit32] Mikhaleva N. S., Visotin M. A., Kuzubov A. A., Popov Z. I. (2017). J. Phys. Chem. C.

[cit33] Chang K., Chen W. (2011). Acs Nano.

[cit34] Ma Y., Dai Y., Wei W., Niu C., Yu L., Huang B. (2011). J. Phys. Chem. C.

[cit35] Wang Y., Jiao Z., Ma S., Guo Y. (2019). J. Power Sources.

[cit36] Bijoy T. K., Sudhakaran S., Lee S. C. (2024). ACS Omega.

[cit37] Wasalathilake K. C., Hu N., Fu S., Zheng J., Du A., Yan C. (2021). Appl. Surf. Sci..

[cit38] Liu A. Y., Wentzcovitch R. M., Cohen M. L. (1989). Phys. Rev. B: Condens. Matter Mater. Phys..

[cit39] Zhang J., Zhang Y. F., Huang S. P., Lin W., Chen W. K. (2019). J. Phys. Chem. C.

[cit40] Kresse G., Hafner J. (1993). Phys. Rev. B: Condens. Matter Mater. Phys..

[cit41] Kresse G., Furthmuller J. (1996). Phys. Rev. B: Condens. Matter Mater. Phys..

[cit42] Grimme S., Antony J., Ehrlich S., Krieg H. (2010). J. Chem. Phys..

[cit43] Zhang Z., Zhang Y., Li Y., Lin J., Truhlar D. G., Huang S. (2018). Chem. Mater..

[cit44] Sheppard D., Terrell R., Henkelman G. (2008). J. Chem. Phys..

[cit45] Tang W., Sanville E., Henkelman G. (2009). J. Phys.: Condens. Matter.

[cit46] Hu W., Wang T., Zhang R., Yang J. (2016). J. Mater. Chem.
C.

[cit47] Li Y., Wu W., Ma F. (2019). J. Mater. Chem. A.

[cit48] Barik G., Pal S. (2021). J. Phys. Chem. C.

[cit49] He X., Tang A., Li Y., Zhang Y., Chen W., Huang S. (2021). Appl. Surf. Sci..

[cit50] Masood M. K., Wang J., Song J. T., Liu Y. (2024). Appl. Surf. Sci..

[cit51] Zheng J., Ren Z., Guo P., Fang L., Fan J. (2011). Appl. Surf. Sci..

[cit52] Li Y., Wu D., Zhou Z., Cabrera C. R., Chen Z. (2012). J. Phys. Chem. Lett..

[cit53] Aydinol M. K., Kohan A. F., Ceder G., Cho K., Joannopoulos J. (1997). Phys. Rev. B: Condens. Matter Mater. Phys..

[cit54] Er D., Li J., Naguib M., Gogotsi Y., Shenoy V. B. (2014). ACS Appl. Mater. Interfaces.

[cit55] Mortazavi B., Dianat A., Cuniberti G., Rabczuk T. (2016). Electrochim. Acta.

[cit56] Sun Q., Dai Y., Ma Y., Jing T., Wei W., Huang B. (2016). J. Phys. Chem. Lett..

