# Supplementary material for: BC2N/graphene heterostructures as anode materials with improved performance for lithium-ion batteries
Source: RSC Adv. 2026 Feb 5;16(9):7673–80. doi: 10.1039/d5ra07205k (PMC12874546; doi:10.1039/d5ra07205k)
Supplement: RA-016-D5RA07205K-s001 [file RA-016-D5RA07205K-s001.pdf]

Supplementary Information (SI) for RSC Advances.
This journal is © The Royal Society of Chemistry 2026

Supporting Information For

BC_2N/graphene heterostructures as anode materials with improved performance for lithium-ion battery

Jing Zhang,[a,*] Zhen Yao,[b] Chaoyan Lou,[c] Liming Zhao,[d] Kuixing Ding,[d] Xiongfeng Ma,[e,*] Wenkai Chen[f,*] Pengyue Zhang,[c] and Miaogen Chen[g]

[a] *College of Science, China Jiliang University, Hangzhou 310018, China*

[b] *Hangzhou Papermate Science &Technology Co., Ltd, Hangzhou 310018, China*

[c] *Management Science and Engineering, China Jiliang University, Hangzhou, 310018, China*

[d] *College of Materials and Chemistry, China Jiliang University, Hangzhou, 310018, China*

[e] *College of Engineering, Xi'an International University, Xi'an, 710077, China*

[f] *Department of Chemistry, Fuzhou University, Fuzhou, Fujian 350116, China*

[g] *College of Science, Zhejiang University of Science and Technology, Hangzhou 310023, China*

*Corresponding authors, E-mail: jingzhang0218@163.com
maxiongfeng1992@163.com
wkchen@fzu.edu.cn

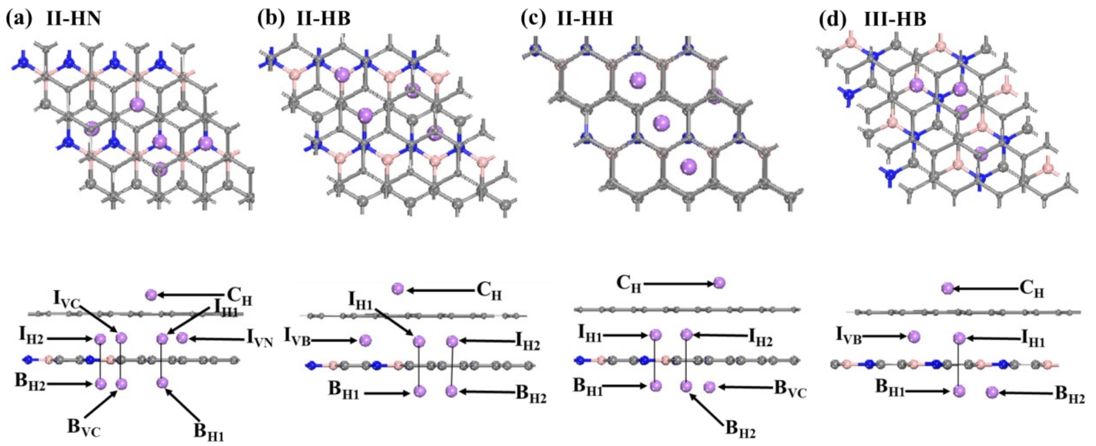

Fig. S1 Top and side views of stable adsorption sites for Li-ion adsorption in Li/G/BC_2N, G/Li/BC_2N, and G/BC_2N/Li of II-HN, II-HB, II-HH, and III-HB.

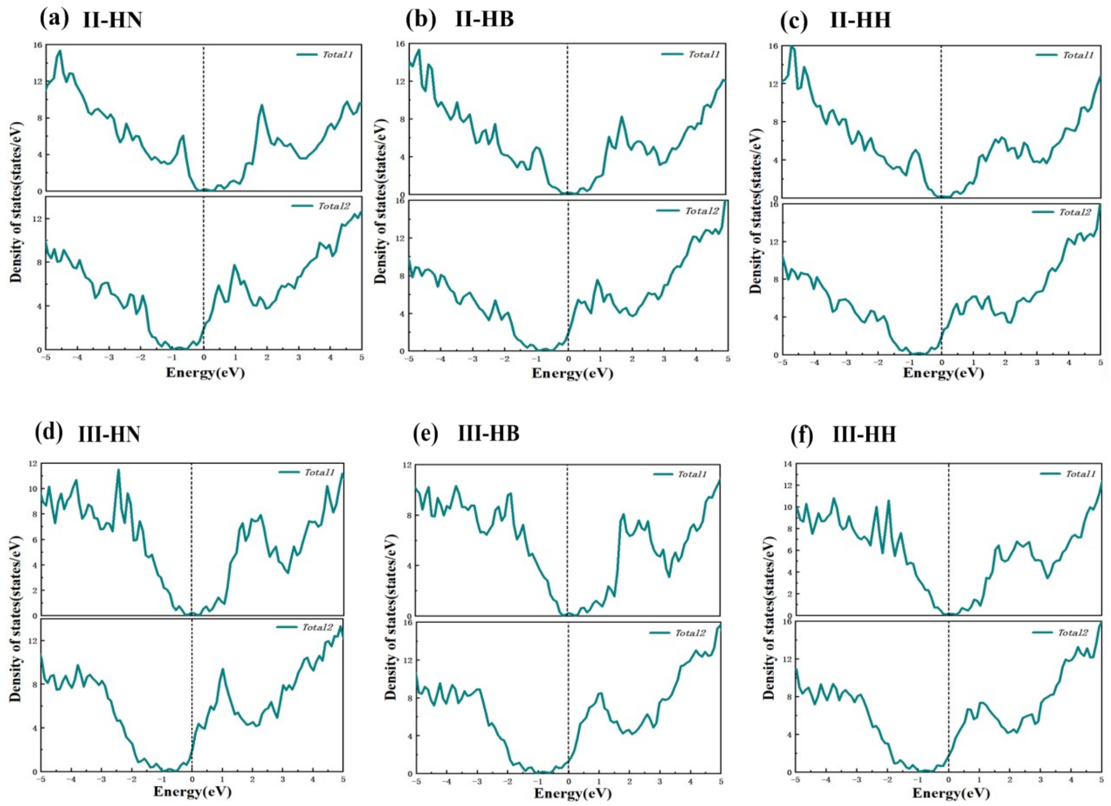

Fig. S2 Total densities of states of II-HN, II-HB, II-HH, III-HN, III-HB, and III-HH heterostructures before and after Li adsorption.

Table S1 Calculated the formation energies (E_{stack}), as well as the total energy of the BC_2N/graphene heterostructures ($E_{BC_2N/G}$), graphene (E_G), and BC_2N monolayer(E_{BC_2N}).

system	E_{stack}(eV)/C atom	E_{stack}(eV)	$E_{BC_2N/G}$(eV)	E_G(eV)	E_{BC_2N}(eV)
**II-HN**	-0.023	-0.736	-580.067	-296.717	-282.614
**II-HB**	-0.045	-1.44	-580.771	-296.717	-282.614
**II-HH**	-0.04	-1.28	-580.583	-296.717	-282.614
**III-HN**	-0.05	-1.6	-571.384	-296.717	-273.067
**III-HB**	-0.02	-0.64	-571.206	-296.717	-273.067
**III-HH**	-0.04	-1.28	-570.777	-296.717	-273.067

Table S2 Calculated adsorption energies (E_{ad}), bader charge transfer (q), and the height between Li atom and monolayer at the more stable adsorption sites, for Li adsorbed on II-HN, II-HB, II-HH, and III-HB heterostructures.

system	Li site	E_{ad} (eV)	q (|e|)	Height (Å)
**II-HN** (BC_2N/Li/G)	I_{H1}	-0.59	0.84	1.41
	I_{H2}	-0.39	0.85	1.53
	I_{VN}	-0.48	0.85	1.62
	I_{VC}	-0.57	0.84	1.63
**II-HN** (Li/BC_2N/G)	B_{H1}	-0.12	0.88	1.67
	B_{H2}	-0.08	0.90	1.66
	B_{VC}	-0.10	0.90	1.77
**II-HN** (BC_2N/G/Li)	C_H	-0.06	0.89	4.94
**II-HB** (BC_2N/Li/G)	I_{H1}	-0.69	0.85	1.64
	I_{H2}	-0.52	0.85	1.57
	I_{VB}	-0.58	0.85	1.57
**II-HB** (Li/BC_2N/G)	B_{H1}	-0.10	0.88	1.67
	B_{H2}	-0.15	0.90	1.68
**II-HB** (BC_2N/G/Li)	C_H	-0.04	0.89	5.04
**II-HH** (BC_2N/Li/G)	I_{H1}	-1.05	0.85	1.68
	I_{H2}	-0.89	0.85	1.70
**II-HH** (Li/BC_2N/G)	B_{H1}	-0.12	0.88	1.68
	B_{H2}	-0.10	0.90	1.69
	B_{VC}	-0.07	0.90	1.75

**II-HH** (BC_2N/G/Li)	C_H	-0.06	0.89	5.13
**III-HB** (BC_2N/Li/G)	I_{H1}	-0.71	0.85	1.73
	I_{VB}	-0.81	0.85	1.83
**III-HB** (Li/BC_2N/G)	B_{H1}	-0.21	0.88	1.7
	B_{H2}	-0.11	0.89	1.77
**III-HB** (BC_2N/G/Li)	C_H	-0.09	0.89	4.87
